# Optoelectronic integration: from enabling technologies to photonic computing and emerging applications

**DOI:** 10.1093/nsr/nwag619

**Published:** 2026-09-29

**Authors:** Jingxuan Chen, Mingjin Wang, Cheng Wang, Xingjun Wang, Daoxin Dai, Wanhua Zheng

**Affiliations:** Key Laboratory of Solid-State Optoelectronics Information Technology, Institute of Semiconductors, Chinese Academy of Sciences, China; Key Laboratory of Solid-State Optoelectronics Information Technology, Institute of Semiconductors, Chinese Academy of Sciences, China; School of Future Technology, University of Chinese Academy of Sciences, China; College of Materials Science and Opto-electronics Technology, University of Chinese Academy of Sciences, China; Department of Electrical Engineering, City University of Hong Kong, China; State Key Laboratory of Terahertz and Millimeter Waves, City University of Hong Kong, China; State Key Laboratory of Photonics and Communications, School of Electronics, Peking University, China; Key Laboratory for Advanced Optoelectronic Integrated Chips of Jiangsu Province, Peking University Yangtze Delta Institute of Optoelectronics, China; Frontiers Science Center for Nano-optoelectronics, Peking University, China; State Key Laboratory for Extreme Photonics and Instrumentation, College of Optical Science and Engineering, International Research Center for Advanced Photonics, Zhejiang University, China; China Jiliang University, China; Ningbo Research Institute, Zhejiang University, China; Key Laboratory of Solid-State Optoelectronics Information Technology, Institute of Semiconductors, Chinese Academy of Sciences, China; School of Future Technology, University of Chinese Academy of Sciences, China; College of Materials Science and Opto-electronics Technology, University of Chinese Academy of Sciences, China

The exponential growth of data and the rapid advancement of artificial intelligence are pushing electronic devices and links toward fundamental physical limits. Bandwidth density, energy efficiency and latency can no longer be improved continuously to satisfy the demands of increasingly powerful computing systems if no fundamentally new approaches are introduced. Optoelectronic integration has emerged as a promising pathway forward by combining the high bandwidth of photonics with the programmability and processing capabilities of electronics. This special topic brings together 13 contributions covering recent progress of devices, materials, architectures and emerging applications in the field of optoelectronic integration. An exclusive interview with Prof. Qionghai Dai from Tsinghua University, an Academician of the Chinese Academy of Sciences (CAS), provides a deep perspective on the key challenges, architectural choices and future directions [1] and sets the context for the research advances featured in this special topic of *National Science Review*.

A central theme of this special topic is the continued development of advanced optoelectronic integration technologies. A comprehensive review co-authored by Bowers *et al.* [2] traces the development of the field from integrated photonic devices to neural-network systems and shows how material platforms, device building blocks and system-level co-design are being brought together. At the platform level, Tsang *et al.* [3] offer a panoramic perspective on photonic integrated circuits and their applications, examining the maturity, capabilities and trade-offs of silicon platforms. Building on these platforms, Zhu’s group, together with coauthors [4], report an optically locked, low-noise photonic microwave oscillator, recognized as an important device that bridges the optical and microwave domains. Meanwhile, Wang *et al.* [5] address one of the key engineering challenges in large-scale integration by demonstrating a multichannel flip-chip packaging platform for thin-film lithium niobate photonics. Together, these contributions illustrate how advances in materials, devices, platforms and packaging technologies are converging to establish the solid foundation for scalable optoelectronic integrated systems.

Leveraging institutional and systematic advantages, the CAS has established forward-looking strategic layouts in optoelectronic interconnection and integrated chip technologies toward next-generation information hardware. CAS research institutes have made substantial breakthroughs in large-scale monolithic optical interconnection, heterogeneous material integration and wafer-scale photonic device fabrication. Cross-collaborations between academia and industry are being advanced to develop high-performance, scalable next-generation optical interconnection systems, providing critical technical foundations for large-scale artificial intelligence computing and high-speed data transmission infrastructures.

With these enabling technologies in place, currently photonic computing for artificial intelligence is becoming one of the most application-driven frontiers in optoelectronic integration. At the architectural level, Dai *et al.* [6] demonstrate 192-dimensional multiplexed photonic computing, achieving a high degree of parallelism through the simultaneous use of multiple optical degrees of freedom. Wang *et al.* [7] present an on-chip all-optical interconnect for deep neural network inference, addressing the electro-optic conversion bottleneck that limits the efficiency of conventional architectures.

Looking ahead, as conventional transistor scaling faces increasing physical and economic constraints, photonic computing could provide particularly promising solutions for workloads that can benefit from parallel processing and efficient data transmission. Yet this still requires continued innovations in materials, devices, architectures and system integration [8]. In this special topic, we present three perspectives from academia and industry, to discuss the future technology trends in photonic computation. Dong *et al.* [9] survey major strategies for photonic AI computing, while Shen, the founder of Lightelligence, and his coauthors [10] discuss the practical breakthroughs needed to move large-scale integrated photonic computing toward deployment. Extending the discussion beyond conventional von Neumann architectures and established accelerator paradigms, Gu *et al*. [11] explore brain-like optical computing systems and consider how light might process information in fundamentally different ways.

The opportunities enabled by optoelectronic integration, however, extend well beyond AI acceleration. The field is also opening up new application spaces in which integrated photonic systems can provide capabilities that are difficult to achieve with electronics alone. Quantum information processing is one such area, with integrated optoelectronic chips offering potential advantages in scalability, stability and functionality. Ren, Wang, Xu, Xie and Gong, together with their coauthors [12], provide a comprehensive review of this emerging landscape, covering single-photon sources, entangled-photon generation and on-chip quantum logic gates. For combinatorial optimization, Li *et al.* [13] examine the optical Ising machine as a specialized computational platform for problems whose complexity grows rapidly on conventional computers but can be naturally mapped onto networks of coupled photonic oscillators. In another direction, Shi *et al.* [14] present a perspective on large-scale photonic integrated circuits for advanced displays, demonstrating how integration technologies are also moving into consumer products. These contributions highlight the breadth of the field and show that optoelectronic integration is becoming the technological underpinning of multiple computational and information-processing paradigms.

The diverse advances presented in this special topic resonate with several principles articulated by Academician Dai in the accompanying interview [1]. He identifies heterogeneous integration as the most practical near-term route forward because it allows different functions to be implemented with the material systems most suited to them. More fundamentally, he questions whether increasing the scale of integration should be treated as an objective in its own right. In his view, the more important question is which applications make integration indispensable. Such applications, particularly those driven by the demands of large-scale AI computing, are now beginning to emerge. He further suggests that functional density and system value, measured in bandwidth density and energy per bit or per operation rather than in component count, should serve as more meaningful measures of technological progress.

In summary, the contributions in this special topic trace a coherent path from enabling technologies and integration platforms to functional computing systems and emerging applications. They also reflect the field’s expanding scope, from attractive optical interconnects to emerging applications like AI acceleration, quantum information processing, combinatorial optimization and advanced displays. A common trend for all these applications is large-scale multifunctional optoelectronic integration beyond individual devices and functions. This transition is being driven by the rapidly growing demands of AI, the maturation of photonic foundry infrastructure and continued progress in heterogeneous integration. The next stage of development will determine whether optoelectronic integration remains primarily a means of achieving faster interconnects or evolves into a transformative platform in its own right.
